# Complete Plastid Genome Sequencing of Four *Tilia* Species (Malvaceae): A Comparative Analysis and Phylogenetic Implications

**DOI:** 10.1371/journal.pone.0142705

**Published:** 2015-11-13

**Authors:** Jie Cai, Peng-Fei Ma, Hong-Tao Li, De-Zhu Li

**Affiliations:** 1 Germplasm Bank of Wild Species, Kunming Institute of Botany, Chinese Academy of Sciences, Kunming 650201, Yunnan, China; 2 School of Life Science, Yunnan University, Kunming 650091, Yunnan, China; 3 Kunming College of Life Science, University of Chinese Academy of Sciences, Kunming 650204, China; Nanjing Forestry University, CHINA

## Abstract

*Tilia* is an ecologically and economically important genus in the family Malvaceae. However, there is no complete plastid genome of *Tilia* sequenced to date, and the taxonomy of *Tilia* is difficult owing to frequent hybridization and polyploidization. A well-supported interspecific relationships of this genus is not available due to limited informative sites from the commonly used molecular markers. We report here the complete plastid genome sequences of four *Tilia* species determined by the Illumina technology. The *Tilia* plastid genome is 162,653 bp to 162,796 bp in length, encoding 113 unique genes and a total number of 130 genes. The gene order and organization of the *Tilia* plastid genome exhibits the general structure of angiosperms and is very similar to other published plastid genomes of Malvaceae. As other long-lived tree genera, the sequence divergence among the four *Tilia* plastid genomes is very low. And we analyzed the nucleotide substitution patterns and the evolution of insertions and deletions in the *Tilia* plastid genomes. Finally, we build a phylogeny of the four sampled *Tilia* species with high supports using plastid phylogenomics, suggesting that it is an efficient way to resolve the phylogenetic relationships of this genus.

## Introduction

*Tilia* L. (basswood or lime-tree) is a genus of the family Malvaceae in the order Malvales, which contains 23 species of deciduous tree disjunctly distributed in the temperate area across Asia, Europe and North America [1–3]. Trees of *Tilia* are ecologically and economically important. Most species are the dominant element in the broad-leaved temperate forests, and many species are used for timber, honey resources and cultivated worldwide for ornamental purpose.

Traditionally *Tilia* has been placed in its own family Tiliaceae [4, 5]. However, molecular evidence strongly supported the polyphyly of the traditionally circumscribed Tiliaceae [6]. A clade of Tilioideae was resolved and *Tilia* was closely related to the genera *Craigia* and *Mortoniodendron* in the family of Malvaceae [7, 8]. Within the Malvaceae family, *Tilia* is a distinct genus characterized by woody habit and paddle-shaped bracts of flowers. However, the taxonomy of *Tilia* is difficult and controversial due to limited taxonomic characters and frequently occurred hybridization [3], and species relationships within the genus are poorly known. Previous molecular studies mainly focused on population genetic analyses of some *Tilia* species employing plastid PCR-RFLP markers [9], random amplified polymorphic DNA (RAPD) markers [10–12], microsatellite markers [13]. Recent molecular phylogenetic studies have used chloroplast regions (*rpL*32-*trnL* and *ndhF*-*rpL*32) or ribosomal internal transcribed spacer (ITS) to reconstruct the phylogeny of selected species in *Tilia*, but without satisfaction [14, 15]. An expanded attempt of seven plastid regions and three low-copy nuclear regions on comprehensively sampled taxa did also not well resolve interspecific relationships of *Tilia* [16]. The age of *Tilia* trees when they begin to flower and produce seed ranges from six to 40 years old [3]. The slow nucleotide substitution rates of *Tilia* may be attributed to their long generation times.

The recent availability of the next-generation sequencing techniques has enabled generating large amounts of sequence data at relatively low cost [17–19]. Whole plastid genome is increasingly used for phylogenetic analyses and has proven to be effective in resolving difficult phylogenetic relationships [20–22]. Plastid genomes of angiosperms are well known to be highly conserved [23, 24]. They usually have a circular structure of two copies of large inverted repeats (IR) separated by small (SSC) and large (LSC) single-copy regions [25]. With a size ranging from 120 to 160 kb in general, they also exhibit highly conserved gene content and order [23, 24].

The number of sequenced plant plastid genomes increased rapidly during last decade due to the establishment of the next-generation sequencing techniques. Within the Malvaceae family, however, there are only two genera *Gossypium* [26, 27] and *Theobroma* [28] having their plastid genomes sequenced to date. At present, there is no complete plastid genome from *Tilia* sequenced despite of its ecological and economic importance.

To better understand the evolution of plastid genome and explore the potential of phylogenomics basing on plastid genome sequence to clarify interspecific relationships in *Tilia*, four representative *Tilia* species were sequenced using next-generation Illumina sequencing-by-synthesis technology. The main purposes of this study are to (1) gain insights into the structure of plastid genome of *Tilia* as well as in Malvaceae; and (2) explore the feasibility of plastid phylogenomics in reconstructing a solid interspecific relationships of the long-lived tree genus *Tilia*.

## Materials and Methods

### Plant material

Four species were chosen as representatives of *Tilia* for plastid genome sequencing. Studied species are commonly found in the public woodland where they are native (Table 1) and no specific collecting permits required for sampling. *T*. *amurensis* is listed in the China’s protected species Grade II, but it is not require permit to collect a few twigs and leaves for vouchers and DNA extraction. Healthy and fresh leaves were collected from a single individual for DNA extraction. The voucher specimens of sampled species were all deposited at the Herbarium of Kunming Institute of Botany, Chinese Academy of Sciences (KUN) (Table 1).

**Table 1:** Sequencing information for the four *Tilia* species used in this study.

Species	Voucher No.	Locality	GenBank numbers	Clean reads	Mean coverage
*T*. *amurensis*	11CS2872	Harbin, Heilongjiang, China	KT894772	5,021,460	282x
*T*. *mandshurica*	11CS2873	Harbin, Heilongjiang, China	KT894773	10,712,702	602x
*T*. *oliveri*	12CS5580	Wushan, Chongqing, China	KT894774	13,141,606	739x
*T*. *paucicostata*	13CS6898	Zhongdian, Yunnan, China	KT894775	5,074,944	285x

### DNA extraction and template amplification

Total genomic DNA was extracted from ~100 mg of leaf material using a modified CTAB method [29, 30], and their quality was assessed by agarose gel electrophoresis. We amplified the entire plastid genome using long-range PCR and 9 primer pairs as described in Yang et al. 2014 [30]. Briefly, amplification was performed using Takara PrimeSTAR GXL DNA polymerase (TAKARA BIO INC.) in 25-μl reaction mixtures with 30–100 ng of DNA template. The PCR amplification conditions were the same as those of Yang et al. 2014 [30]. All amplifications were successful and amplicon DNA concentrations were determined by visual approximation using gel electrophoresis. Subsequently, the 9 long-ranged PCR products were pooled together in roughly equal mass mixtures for genome sequencing.

### Illumina sequencing, assembly, and annotation

Pooled amplified plastid DNAs (6 μg) were sheared for short-insert (500 bp) sequencing libraries construction according to the manufacturer’s instructions (Illumina). The 90 bp paired-end reads were generated on an Illumina Hiseq 2000 at BGI Shenzhen, China. Illumina raw reads were first quality trimmed using NGS QC Tool Kit [31] (cut-off value for percentage of read length = 80 and for Phred quality score = 30). The plastid genomes from the filtered clean reads were assembled using the CLC Genomics Workbench v. 6.5 (CLC Bio) de novo assembly program at hash length of 63 and with a minimum contig length of 1 kb. The assembled contigs were analyzed by a BLAST of the nucleotide database at the NCBI (http://www.ncbi.nlm.nih.gov/), and these aligning to the published plastid DNA sequences were collected for genome finishing. The annotation of completed plastid genomes was carried out using the program DOGMA [32]. We then manually adjusted the start and stop codons and intron/exon boundaries if necessary. The annotated GenBank files were used to draw the circular plastid genome maps using the OrganellarGenomeDRAW (OGDRAW) [33].

### Comparison of *Tilia* plastid genome with other Malvaceae genera

The complete plastid genomes of *Gossypium hirsutum* (GenBank NC_007944) and *Theobroma cacao* (GenBank HQ336404) were downloaded from NCBI for comparison. The general plastid genome characters were compared between these two and our sequenced four *Tilia* species. Any large structural events such as gene order rearrangement and IR expansion/contraction were recorded. To investigate the difference in genome size between *Gossypium* and *Tilia*, the plastid DNA sequences of *G*. *hirsutum* and *T*. *mandshurica* were partitioned into genes, introns and intergenic spacers and then were compared separately.

### Sequence analysis for *Tilia* species

The plastid genome sequences of four *Tilia* species and *G*. *hirsutum* were aligned using MAFFT v. 7.215 [34] in the default sets and manually adjusted in MEGA 5.0 [35]. Sequence divergence between the four *Tilia* plastid genome sequences was calculated as uncorrected p-distance using MEGA 5.0. The percentage of variable characters for each noncoding region with an aligned length >200 bp in the genome was calculated as described in Zhang et al [17]. We scored the number of transitional and transversional substitutions among the four *Tilia* plastid genome sequences and decided the direction of substitutions using *G*. *hirsutum* as outgroup. The indels among them were polarized into insertions and deletions in the same way.

### Phylogenetic inference

We downloaded the plastid genome sequences of two species of Brassicaceae, *Arabidopsis thaliana* (GenBank NC_000932) and *Brassica napus* (GenBank NC_016734) as outgroups. The ingroup taxa included one *Theobroma* species, two *Gossypium* species (the second being *G*. *herbaceum* with GenBank NC_016734) and four *Tilia* species sequenced here. The orientation of the SSC regions from two Brassicaceae species and *Theobroma cacao* were manually reversed for alignment. The alignment of nine plastid genomes with one IR region removed was conducted with MAFFT v. 7.215 [34] in the default sets, followed by manual adjustments in MEGA 5.0 [35]. Phylogenetic analysis using maximum likelihood (ML) method was performed using RAxML v. 8.0.20 [36]. Both the unpartitioned and partitioned ML analyses were performed with the dataset dividing into three partitions corresponding to the LSC, SSC and IR region of the plastid genome. The ML tree was constructed with the combined rapid bootstrap of 500 replicates and search for the best tree in a single run under the GTR + G model as suggested in the RAxML manual. Bayesian analysis was performed using MrBayes v. 3.2 [37] with the GTR + I +G model in the unpartitioned way. The Markov chain Monte Carlo (MCMC) algorithm was run for two million generations with trees sampled very 100 generations. The convergence was reached with the average standard deviation of split frequencies (ASDFs) following 0.01. The first 25% of trees generated were discarded as burn-in and the remaining trees were used to build majority-rule consensus tree. To test the rate of evolution of *Tilia* relative to other Malvaceae species we applied Tajima relative rate test [38] on the plastid genome sequence alignment. The relative rates of evolution between each of the four *Tilia* species and *G*. *hirsutum* were evaluated using the *Theobroma cacao* as outgroup.

## Results

### Sequencing of *Tilia* plastid genomes

**Fig 1:**
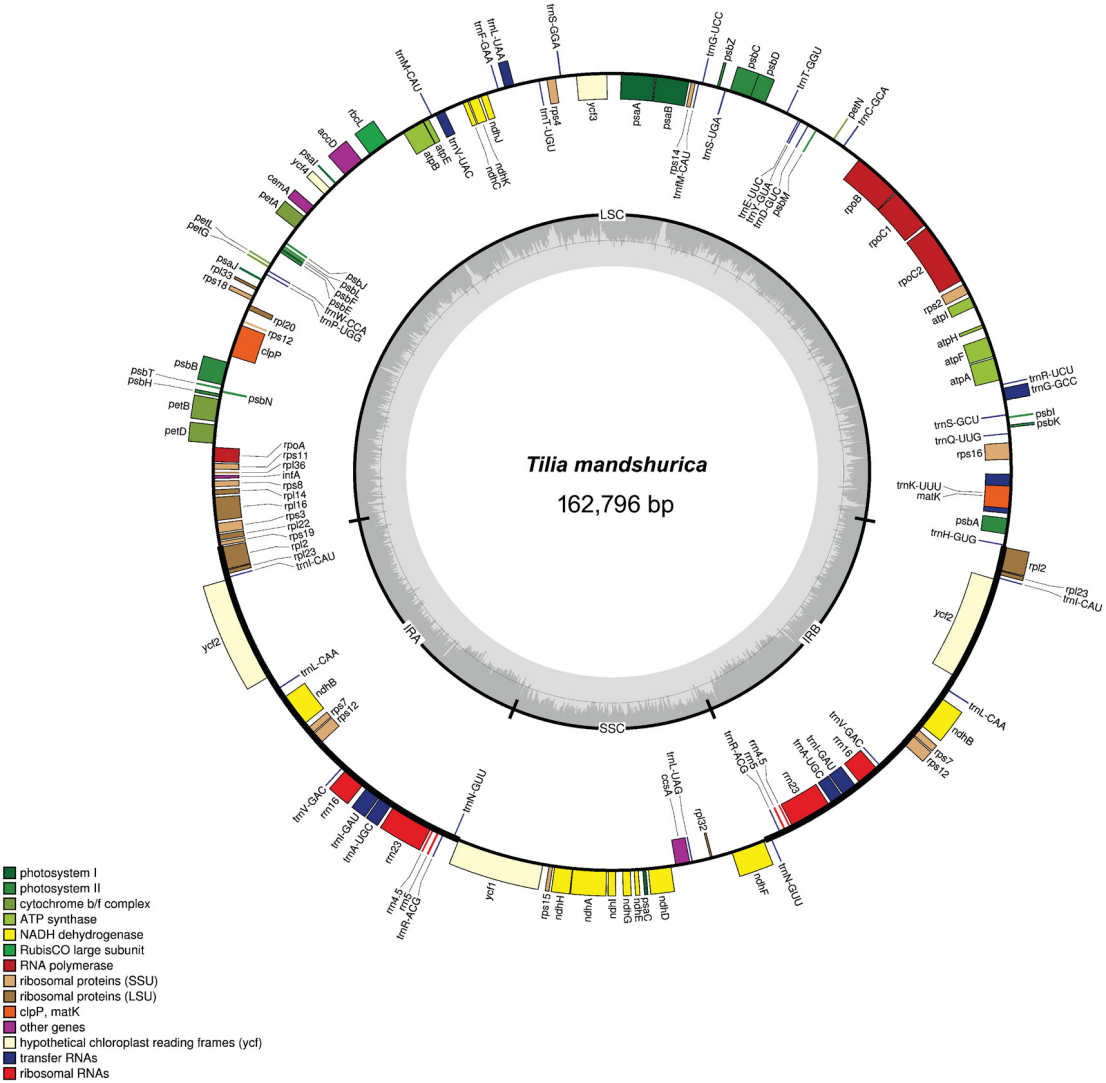
Circular gene map of the plastid genome of *Tilia mandshurica*. Genes drawn within the circle are transcribed clockwise, while those drawn outside are transcribed counterclockwise. Genes are color-coded according to their functional groups.

Illumina 90-bp paired-end sequencing of long-rang PCR amplified plastid DNA generated 5,021,460–13,141,606 clean reads for the four sampled *Tilia* species, with an average sequencing depth from 282× to 739× (Table 1). Using the combination of de novo and reference-guided assembly, we obtained the complete plastid nucleotide sequences for all four species. The determined nucleotide sequences of the four plastid genomes range narrowly from 162,653 bp in *Tilia paucicostata* to 162,796 bp in *Tilia mandshurica* (Table 2). They all have a genome structure resembling those of the vast majority of angiosperms, consisting of a pair of IRs separated by LSC and SSC, and the gene map of *T*. *mandshurica* plastid genome is presented in Fig 1 as a representative. The four genomes encode an identical set of 130 genes, of which 113 are unique and 17 are duplicated in the IR regions (Table 2), and the arrangements of these 130 genes in them are totally collinear. The 113 unique genes include 79 protein-coding genes, 30 tRNA genes and 4 rRNA genes. They also have an identical GC content of 36.5% that is similar to other angiosperms plastid genomes [23, 24].

**Table 2:** Comparison of Malvaceae plastid genomes sampled in this study.

	*Gossypium hirsutum*	*Theobroma cacao*	*Tilia amurensis*	*Tilia mandshurica*	*Tilia oliveri*	*Tilia paucicostata*
**Size (bp)**	160,301	160,604	162,715	162,796	162,734	162,653
**LSC (bp)**	88,816	89,395	91,124	91,127	91,095	91,139
**SSC (bp)**	20,269	20,187	20,397	20,371	20,381	20,380
**IR (bp)**	25,608	25,511	25,597	25,649	25,629	25,567
**Number of protein-coding genes** ^a^	84 (6)	85 (6)	85 (6)	85 (6)	85 (6)	85 (6)
**Number of tRNA genes** ^a^	37 (7)	37 (7)	37 (7)	37 (7)	37 (7)	37 (7)
**Number of rRNA genes** ^a^	8 (4)	8 (4)	8 (4)	8 (4)	8 (4)	8 (4)
**GC content (%)**	37.3	36.9	36.5	36.5	36.5	36.5

^a^The numbers in parenthesis indicate the genes duplicated in the IR regions.

### Comparisons with other Malvaceae plastid genomes

The comparisons of general genomic features between *Gossypium hirsutum* [26] as a representative species from genus *Gossypium*, *Theobroma cacao* [28] and four sequenced *Tilia* species were presented in Table 2. All plastid genomes except for *G*. *hirsutum* share identical complements of coding genes with similar order. The *infA* gene in the plastid genome of genus *Gossypium* was inferred to be pseudogene due to the presence of frameshift indels [26]. In addition, the GC content of Malvaceae species is almost the same (Table 2).

All plastid genomes of Malvaceae possess a typical quadripartite structure of angiosperms. However, the SSC region in the assembled plastid genomes of *Gossypium* and *Tilia* is in the reverse orientation relative to *Theobroma* and most other angiosperms. The four *Tilia* species have the same IR and SSC boundary with 36-bp sequence of the *ycf1* gene extending into the IR regions. The *ycf1* gene also extended into the IR regions with 98-bp sequence duplication in *G*. *hirsutum*. However, this gene is wholly confined in the SSC region in *Theobroma cacao* while the *ndhF* gene had 6-bp sequence duplicated in the IR regions.

**Fig 2:**
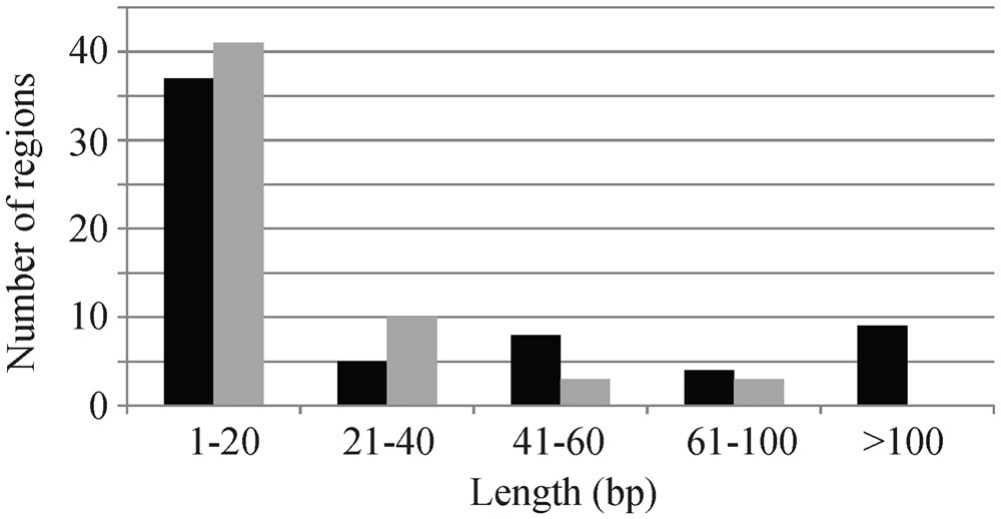
The number of noncoding regions with different sizes between the plastid genomes of *Tilia mandshurica* and *Gossypium hirsutum*. The black bar indicates the regions are larger in *T*. *mandshurica*, while the gray bar indicates the regions are larger in *G*. *hirsutum*.

In terms of genome size, the plastid genome of *Tilia* (162,653–162,796 bp) is slightly larger than those of *Gossypium* (159,039–160,433 bp) and *Theobroma* (160,604 bp). We selected *T*. *mandshurica* and *G*. *hirsutum* as representative species to investigate the trend toward increased genome size in *Tilia*. The whole plastid genome of *T*. *mandshurica* is 2495 bp larger than that of *G*. *hirsutum*, and all three regions LSC, SSC and IR of *T*. *mandshurica* are larger with LSC (2311 bp) accounting for most variation in genome size (Table 2). Among the 78 common unique protein-coding genes between these two genomes there are 11 genes with difference in length. Four genes (*atpI*, *ccsA*, *rbcL*, and *ycf1*) are larger in *T*. *mandshurica* while seven genes (*accD*, *matK*, *ndhF*, *petB*, *rpl22*, *rpoA*, and *rpoC2*) are larger in *G*. *hirsutum*, accounting for 63 bp and 180 bp of the variation in genome size respectively. On the other hand, the majority of noncoding (intergenic and intron) regions (120 of 153) show variations in length. The number of noncoding regions that are larger is similar in *T*. *mandshurica* and *G*. *hirsutum* (63 versus 47) (Fig 2). Nevertheless, the 9 regions with length difference above 100 bp are all those larger in *T*. *mandshurica* than *G*. *hirsutum*. These 9 regions are *ycf3-trnS-GGA*, *ndhC-trnV-UAC*, *trnH-GUG-psbA*, *trnT-UGU-trnL-UAA*, *trnR-UCC-atpA*, *psbZ-trnG-UCC*, *atpB-rbcL*, *trnC-GCA-petN*, and *trnK-UUU-rps16*. These length differences are mainly caused by the large insertions in the *T*. *mandshurica* (or deletions in *G*. *hirsutum*) plastid genome and there are 8 indels (insertions or deletions) in all larger than 100 bp. The total length of these indels is 1990 bp, explaining ~80% of variation in genome size.

### Sequence divergence of *Tilia* plastid genomes

**Fig 3:**
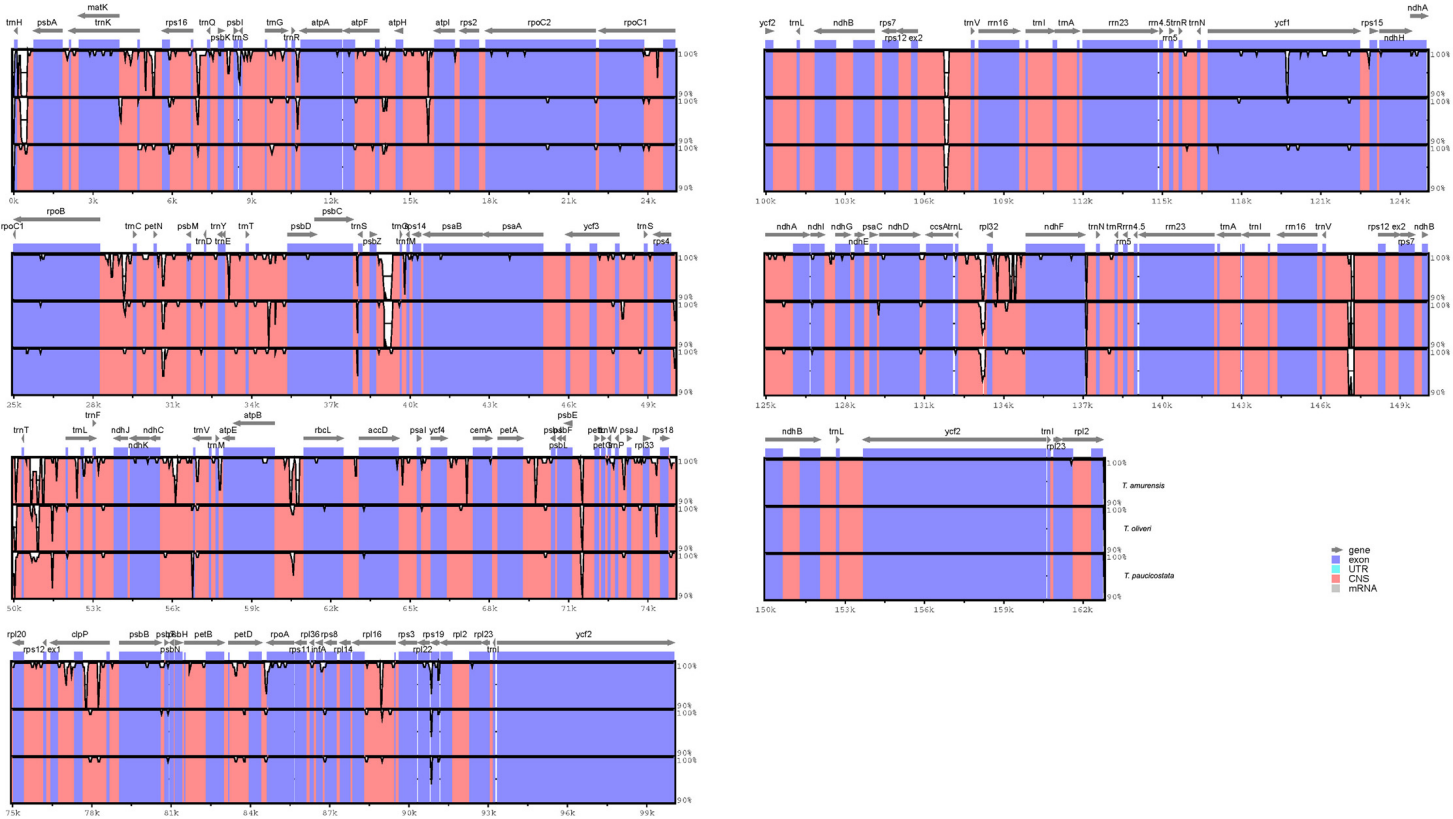
mVISTA percent identity plot comparing the four *Tilia* plastid genomes with *T*. *mandshurica* as a reference. Vertical scale indicates the percentage of identity ranging from 70% to 100%. Coding regions are in blue and noncoding regions are in pink.

**Fig 4:**
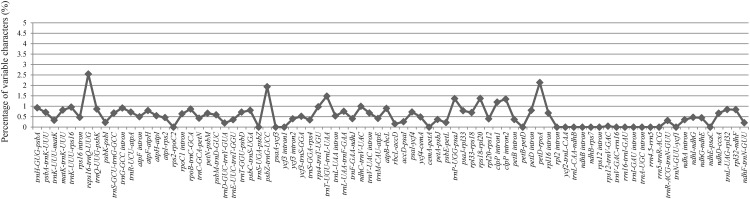
Percentage of variable characters in aligned noncoding regions of the four *Tilia* plastid genomes. These regions are oriented according to their locations in the plastid genome.

We plotted sequence identity among the four *Tilia* plastid genomes using the mVISTA software [39] with *T*. *mandshurica* as a reference. A genome-wide alignment reveals globally high sequence similarity (> 90% identity) among them (Fig 3). The overall sequence divergence estimated by p-distance among the four genomes was only 0.0013. The pairwise p-distance between the four species ranged from 0.0004 to 0.0021, and the *T*. *amurensis* has a somewhat larger sequence divergence from the others within the genus. We also compared the sequence divergence among the different noncoding regions in the four *Tilia* species. Among the 84 noncoding regions, the percentage of variation ranged from 0 to 2.55%, and there were no mutational hotspots identified (Fig 4).

**Fig 5:**
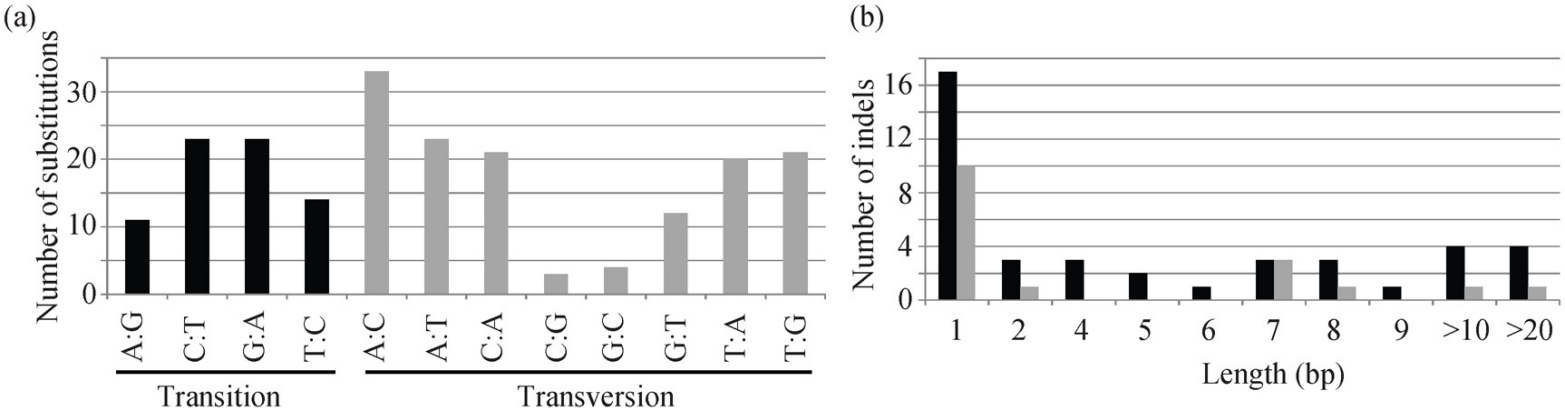
Nucleotide sequence variations identified in the four *Tilia* plastid genomes. **(A)** The nucleotide substitution patterns in the plastid genomes. **(B)** The length distribution of insertions (black) and deletions (gray) in the plastid genomes.

We further investigated the pattern of sequence divergence in these four plastid genomes. Using *G*. *hirsutum* as an outgroup, we only considered these substitutions for which the direction could be unambiguously identified and there were a total of 208 substitutions determined in the whole genome (Fig 5A). Among them, there were 71 transitions and 137 transversions and the transition/transversion ratio (Ts/Tv = 0.52) was nearly identical to the 1:2 ratio expected from equal rates of transition and transversion. More specifically, we observed a significant excess of A to C and lack of G to C and C to G transversions relative to all other substitutions (Fig 5A). The insertions or deletions in plastid genomes of *Tilia* were also inferred with *G*. *hirsutum* as an outgroup. The number of insertions (41) is much larger than the number of deletions (17) (Fig 5B). And about 41% of the total insertions and 59% of the total deletions are of size 1 bp. The longest insertion and deletion was 76 and 21 bp, respectively. In addition, 52 of these 58 indels were associated with tandem repeats.

### Phylogenetic analyses of *Tilia*

**Fig 6:**
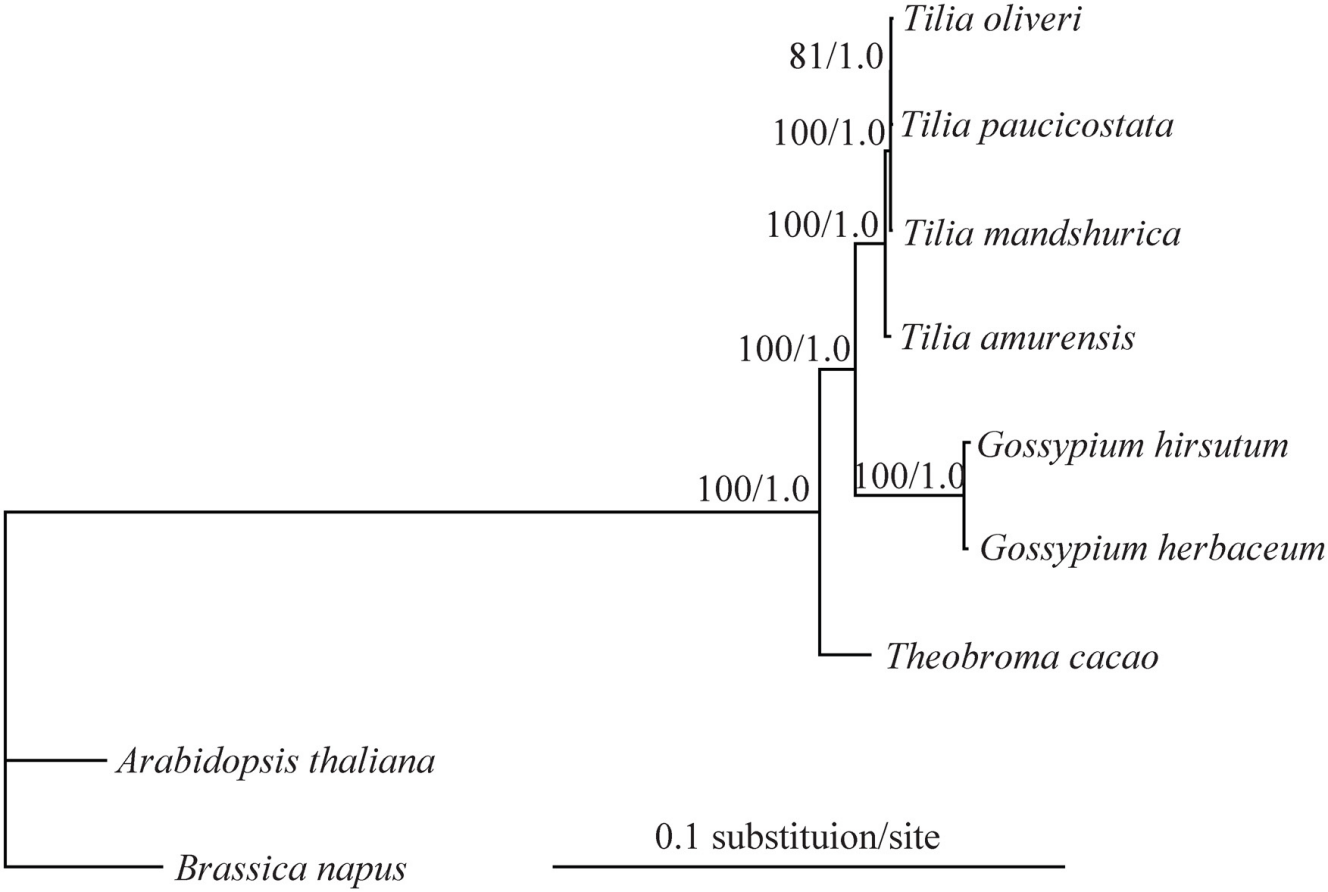
Maximum likelihood phylogeny of the seven Malvaceae species based on the complete plastid genome sequences. The numbers associated with the nodes are bootstrap support and posterior probability values.

The data matrix for phylogenetic analyses contained the whole plastid genome with one IR region removed for nine taxa, including six species of Malvaceae and two outgroups from Brassicaceae. The data set comprised of 145,679 nucleotide positions with 3,377 informative sites for the ingroup taxa. However, there were only 20 informative sites for the four *Tilia* species. Unpartitioned ML analyses resulted in a fully resolved tree with 5 of the 6 nodes supported by 100% bootstrap values (Fig 6). ML analyses partitioned by the three plastid genomic regions (LSC, SSC, and IR) yielded an identical topology with the same 5 nodes 100% supported (data not shown). The remaining one received 81% and 82% bootstrap values from unpartitoned and partitioned analyses, respectively. In Bayesian analysis, the identical topology was obtained with a posterior probability (PP) of 1.0 for all nodes (Fig 6). *T*. *amurensis* was sister to the other three sampled *Tilia* species, and *T*. *mandshurica* was then sister to the grouping of *T*. *oliveri* and *T*. *paucicostata* (Fig 6).

As can been seen in Fig 6, the branch length leading to *Tilia* and especially those among species were extremely short. This indicated that *Tilia* likely had a slow rate of evolution relative to *Gossypium*. To explore the rate heterogeneity existed between *Gossypium* and *Tilia*, we applied Tajima relative rate test [38] on the sequence alignments of whole plastid genome. This test showed that all the four sampled *Tilia* species were evolving significantly slower than the *Gossypium* (*p* < 0.001; Table 3).

**Table 3:** Results of Tajima relative rate test.

Ingroup1	Ingroup2	Outgroup	Identical sites	Divergent sites	Ingroup1 specific^a^	Ingroup2 specific	Outgroup specific	Chi-square statistic	p-value	Slow
*Gossypium hirsutum*	*Tilia mandshurica*	*Theobroma cacao*	125,627	113	2456	725	1801	941.96	< 0.001	*Tilia mandshurica*
*Gossypium hirsutum*	*Tilia paucicostata*	*Theobroma cacao*	125,624	112	2458	727	1801	940.77	< 0.001	*Tilia paucicostata*
*Gossypium hirsutum*	*Tilia amurensis*	*Theobroma cacao*	125,670	104	2452	694	1813	982.38	< 0.001	*Tilia amurensis*
*Gossypium hirsutum*	*Tilia oliveri*	*Theobroma cacao*	125,625	112	2451	720	1804	944.93	< 0.001	*Tilia oliveri*

^a^ These nucleotide sites were identical in ingroup2 and outgroup but not in ingroup1, and the same idea applies for ingroup2 and outgroup specific.

## Discussion

### Comparison of plastid genome within Malvaceae

In this study, we determined the complete plastid genome sequences from four *Tilia* species using Illumina sequencing technology. In addition to *Gossypium* and *Theobroma* [26–28], *Tilia* is the third genus within the family Malvaceae to have its complete plastid genome sequenced. All of the four *Tilia* plastid genomes possess a typical quadripartite structure of angiosperms with a pair of inverted repeats dividing the whole genome into two single copy regions (Fig 1). In comparison to *Gossypium* and *Theobroma*, no significant structural reconfigurations such as inversions or gene relocations were detected in the four *Tilia* plastid genomes. The six plastid genomes analyzed here are rather conserved, and only with minor variations in the junctions between the SSC and IRs regions, which are usually different within the same family [40–42]. The four *Tilia* plastid genomes have the same boundary between the SSC and IRs regions with 36-bp sequence of the *ycf1* gene duplicated in IRs and the length of duplication is 98 bp in *G*. *hirsutum*. In the *Theobroma cacao* plastid genome, it is the *ndhF* gene rather than *ycf1* extending into the IRs. However, the SSC region in the *Tilia* plastid genomes was assembled to being in the reverse orientation relative to *Theobroma* and majority of angiosperms while identical to *Gossypium*. The SSC region could exist in two orientations in plastid genomes [43, 44] and this result does not reflect any differences in gene order in these genomes.

In terms of gene content, the *Tilia* plastid genomes share the same set of 85 protein genes with *Theobroma* rather than *Gossypium* (Table 2), although *Tilia* has a closer relationship to *Gossypium* (Fig 6). The *infA* gene that has become a pseudogene in *Gossypium* has intact reading frame and can be functional in *Tilia*. This result indicates that pseudogenization of *infA* independently occurred in the *Gossypium* lineage. In terms of genome size, the *Tilia* plastid genomes show a trend toward increased size within Malvaceae. We divided the whole plastid genome into genes and intergenic spacers and compared their lengths between *Tilia* and *Gossypium*, finding that the variation in genome size is mostly due to length differences in the noncoding regions (Fig 2). Furthermore, a few large indels (> 100 bp) instead of many small indels can be responsible for ~80% of the total length difference between these two genera.

### Molecular evolution of *Tilia* plastid genome sequences

In addition to the rather conserved evolution of genome structure, the genetic divergence is extremely low among the four *Tilia* plastid genomes, and there is no mutation hotspot region identified across the genome (Figs 3 and 4) as in other angiosperms [17, 45, 46]. Within the four species, the *T*. *amurensis* is slight divergent relative to the others. Among a total of 208 substitutions identified among the four *Tilia* plastid genomes (Fig 5A), the transition/transversion ratio of 0.52 is very close to the expected 1:2 ratio in considering equal rates of transition and transversion. The rate of transitions is not significantly elevated as demonstrated recently in many other plant plastid genomes [22, 27, 47]. However, there are a significant excess of A to C and lack of G to C and C to G transversions among the eight types of transversions (Fig 5A). The underlying mechanism under this phenomenon would require more studies to clarify in the future.

In addition to substitutions, the indels are another important class of genetic variation [48–50]. The insertions occur much more frequently than the deletions in the *Tilia* plastid genomes (Fig 5B), consistent with the trend toward larger genome size within Malvaceae. Approximately half of the total indels are of size 1 bp and mainly occur on the homopolymer regions, and almost entirely of the remaining indels > 1 bp are associated with tandem repeats. This result indicates that these indels very likely originated as mutation events formed by slipped strand mispairing [51].

### Using plastid phylogenomics to resolve phylogeny of *Tilia*

As suggested by the low sequence divergence observed among the four *Tilia* plastid genomes, the slowdown in evolutionary rates may occur in this long-lived tree genus. Relative rate test does demonstrate a reduced rate of evolution in the *Tilia* relative to its relative genus *Gossypium* (Table 3). The slow evolutionary rate in *Tilia* can be largely attributed to its long generation times [3]. Although there are a few phylogenetic informative sites contained in the *Tilia* plastid genomes, the phylogeny of the four sampled *Tilia* species is well resolved (Fig 6). As previous studies employing multi nuclear and plastid DNA regions have failed to reconstruct the phylogeny of *Tilia* [14, 16], our successful reconstruction of phylogeny for the four *Tilia* species sampled here indicates that plastid phylogenomics holds promise in resolving the interspecific relationships of this ecologically and economically important genus.
